# Number of Medical Journals

**Published:** 1856-12

**Authors:** 


*Number of Medical Journals.*

A recent number of the *Memphis Medical Recorder*, gives the following statistics in relation to the number of Medical Journals published in the different countries of Europe and America, viz.:—

"Germans 58, Dutch 8, Swedish 18, English 30, French 34, Belgian 13, Italian 12, Spanish 9, Canadian 3, United States 40—total, 225. The Journals of Pharmacy are included in this list, of which there is a goodly number on both sides of the Atlantic; but it does not embrace the numerous journals, a large majority of which are published in this country, devoted to some one or another of the exclusive systems of practice in vogue."

